# Scale-dependent power law properties in hashtag usage time series of Weibo

**DOI:** 10.1038/s41598-023-49572-6

**Published:** 2023-12-15

**Authors:** Jiwei J. Jiang, Kenta Yamada, Hideki Takayasu, Misako Takayasu

**Affiliations:** 1 School of Computing, Tokyo Institute of Technology, 4259 Nagatsuta-cho, Midori-ku, Yokohama, 226-8502 Japan; 2 Faculty of Global and Regional Studies, University of the Ryukyus, Nishihara, Okinawa 903-0213 Japan; 3 Sony Computer Science Laboratories, 3-14-13 Higashi-Gotanda, Shinagawa-ku, Tokyo 141-0022 Japan

**Keywords:** Applied physics, Applied mathematics, Statistics, Complex networks, Statistical physics, Phase transitions and critical phenomena

## Abstract

We analyze the time series of hashtag numbers of social media data. We observe that the usage distribution of hashtags is characterized by a fat-tailed distribution with a size-dependent power law exponent and we find that there is a clear dependency between the growth rate distributions of hashtags and size of hashtags usage. We propose a generalized random multiplicative process model with a theory that explains the size dependency of the fat-tailed distribution. Numerical simulations show that our model reproduces these size-dependent properties nicely. We expect that our model is useful for understanding the mechanism of fat-tailed distributions in various fields of science and technology.

## Introduction

Fat-tailed distributions are widely observed in nature and man-made phenomena. As the name implies, they are a kind of probability distributions that have a slower decay on the tail than the normal distribution or the exponential distribution. There are many distributions belonging to the class of fat-tailed distributions, among them, the power law (or Pareto) distribution is the most easily recalled, and there are others such as the log-normal distribution, the stretched exponential distribution, and so on. The power law distribution has been attracted the attention of many researchers in various fields, such as the fluctuation of market price in Economics^1^, phase transitions and critical phenomena in Physics^2^, and scale-free degree distribution^3^ in network science. The formation mechanism of power-law distributions is explained by various mathematical and physical models^1–18^ for example, the stable distributions theory of sums of random variables^8^, the maximization of generalized entropy in Tsallis statistics^4^, and the superposition of basic probability distributions, also known as the superstatistics theory^5–7^, and so on.

For time series data, the random multiplicative process model^12,19–23^ is widely known to explain the mechanism of the formation of power law distributions. It is well-known that a simple multiplicative stochastic process causes a non-stationary log-normal distribution with monotonically changing variance, which is traditionally known as the Gibrat process^24^. By adding an additive noise term^12,19–21^, introducing a reflection wall^22^ or resetting events^23^, the random multiplicative process realizes a stationary distribution with asymptotic power law tails. In this model, the multiplicative stochastic variable has the meaning of growth rate, and it is known that the power law exponent is determined uniquely from the distribution of growth rate by solving the equation $\langle b^\alpha \rangle = 1$, where *b* denotes growth rate, $\langle \cdot \rangle$ represents the average, and $\alpha$ is the power law exponent^12^. There have been many studies on the growth rates of business firms, and a typical growth rate distribution is known as the Laplace distribution which is also called the tent-shaped distribution^25–30^. Similar statistical properties of growth rates are also found in other fields of sciences^31–36^, such as microbial communities, tropical forests, and urban populations^33^, implying that the growth rate statistics show universal properties.

Although the random multiplicative process is plausible from a theoretical viewpoint, there are many cases in real-time series data that some observed fat-tailed distributions are not simply characterized by a power law distribution. In other words, there are cases in which the slope of the log-log plot of cumulative distribution functions is not approximated by a straight line. Thus, it is reasonable to introduce a more general model that can explain the whole fat-tailed distribution.

A recent study on hashtags on Twitter reported that the distribution of daily hashtag usage follows a fat-tailed distribution approximated by a generalized log-normal distribution^37^. In this paper, we collected hashtag usage data on Weibo, which is a mainstream social media in China similar to Twitter, and analyzed the statistical properties of hashtag usage and its growth rate. We observe a fat-tailed distribution with scale-dependent power law properties and introduce a generalized random multiplicative model with additive noise to explain the scale-dependent properties theoretically. In the following, the main results are described in Results, and we introduce details of the simulation experiments in Methods.

## Results

### Time series of hashtag numbers

We analyze the posting behavior of hashtags from Weibo, a mainstream micro-blog social media in China. We collect Weibo data through the publicly available API. Due to the huge volume of users and the limitation of the API, it is impossible to collect all the data for analysis, so we focus on the hashtag posting behavior of approximately about 300,000 users. We collect micro-blogs posted by these users from July 21st to August 18th, 2021. Finally, we extract the hashtags from these micro-blogs, obtaining the time series of hashtag usage count. There are approximately 60,000 different hashtags, the duration of which ranges from 1 to 29 days (the longest observation interval). For convenience of the analysis, we choose 5805 hashtags that were used every day during the observation interval as the object of analysis. The detailed process of collecting hashtag number series data is shown in described in chapter 1 of Supplementary Information.

**Figure 1:**
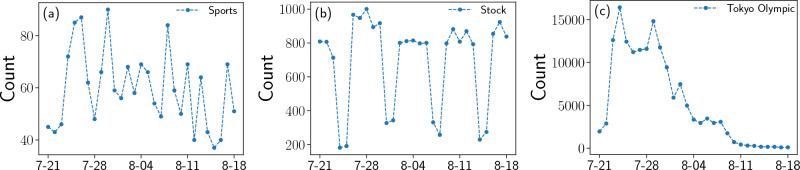
Examples of hashtag usage count time series. The horizontal axis is the date, from July 21st to August 18th (total 29 days), and the vertical axis is the usage count of each hashtag. (**a**) Series of the hashtag “Sports”, which shows a weak stationary pattern; (**b**) series of the hashtag “Stock”, which shows a periodic pattern; (**c**) series of the hashtag “Tokyo Olympic”, which is neither stationary nor periodic.

By comparing the auto-correlation functions of the original and shuffled time series, we simply divide the 5,805 series into three types: weak stationary, periodic, and other. Figure 1 presents examples of these time series.

Although the hashtag numbers time series exhibit different patterns, we analyze them as a whole and observe macroscopic properties. We label hashtag series from 1 to 5805 and define the usage count of hashtag *i* on day *t* as $x_i(t),\ i= 1,2,..., 5805;\ t=1,2,...,29$.

**Figure 2:**
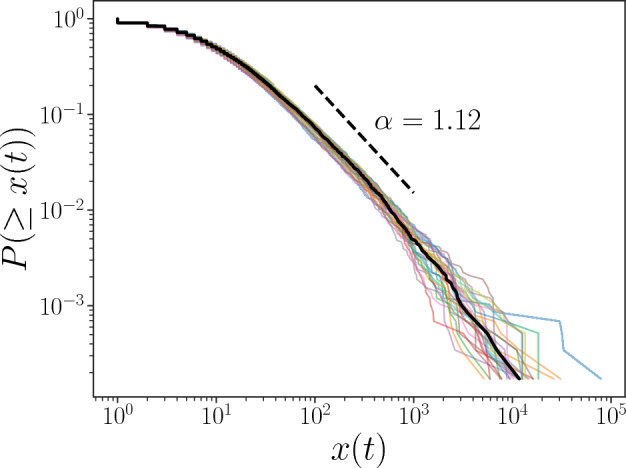
Cumulative distribution function of the count of hashtag usage *x* (*t*). As *t* ranges from 1 to 29, there are 29 cumulative distribution functions plotted as colored solid lines; the median line of these lines is plotted as a black solid line. The black dashed line refers to the slope of the power law distribution whose exponent equals 1.12.

We observe the cumulative distributions of hashtag numbers, $x_i(t)$, for each day *t* and find that the distributions are nearly stationary, following a fat-tailed distribution, as shown in Fig. 2. *x*(*t*) follows a typical fat-tailed distribution with a decay approximated by a power law. For estimation of the power law exponent, we calculate the maximum likelihood estimation to the median line (black line) for $x(t)\ge 10^2$ and find that *x*(*t*) is close to a power law distribution with an exponent close to 1.12, i.e., $P(\ge x(t))\propto x^{-1.12}$. This distribution of *x*(*t*) looks consistent with the result of a former study on hashtag data for the case of Twitter^37^.

**Figure 3:**
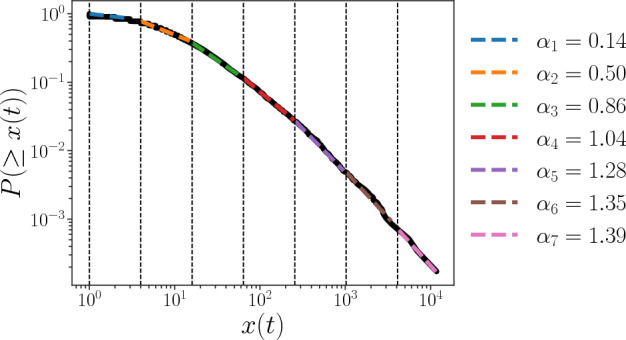
Slope of the cumulative distribution function of the count of hashtag usage *x* (*t*). Black solid line is the median line of the cumulative distributions of *x*(*t*) for different days *t*. *x*(*t*) is divided into seven intervals growing in powers of 2, i.e., $[2^0, 2^2), [2^2, 2^4), [2^4, 2^6), [2^6, 2^8), [2^8, 2^{10}), [2^{10}, 2^{12}), [2^{12}, +\infty )$. The vertical dashed lines refer to the boundaries of these intervals. In each interval, the colored dashed lines are straight lines, the slopes of which are calculated by linear regression of the distribution of *x*(*t*), the absolute values of the slopes are shown in the legend.

As we are concerned with the slope of the fat-tailed distribution of *x*(*t*), we divide *x*(*t*) into seven intervals growing in powers of 2 and perform linear regressions on the median line of the cumulative distribution of *x*(*t*) to calculate the value of the slope, the result is shown in Fig. 3. Between the vertical dashed lines are the intervals in which we divide *x*(*t*). We find that the absolute value of the slope of the distribution, $\alpha _i,\ i=1,2,...,7$, changes in different intervals, which we call scale-dependent power law properties.

### Dynamic properties

Next, we investigate the dynamic properties of the hashtag usage *x*(*t*) through the growth rate *b*(*t*), defined as follows for $x(t)\ne 0$:

1 $$\begin{aligned} b(t) = \frac{x(t+1)}{x(t)}, \end{aligned}$$

**Figure 4:**
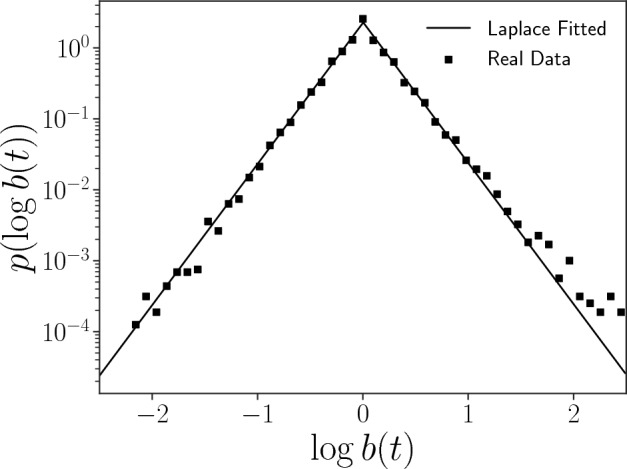
Probability density function of the logarithm of the growth rate of hashtag usage count $\log b(t)$. The probability density is plotted in the log scale of the vertical axis with base 10. The squares show the observed PDF values of the logarithm growth rate of hashtags, fitted with a theoretical Laplace distribution plotted by the straight lines.

We pay attention to 5805 hashtags that were non-zero for all observation days. We plot the probability density function of $\log b(t)$, $p(\log b(t))$, in Fig. 4. We find that the probability density in the log scale is fitted well by a tent-shaped distribution, i.e. a Laplace distribution with $\mu _{\log b(t)}\approx 0$, $\sigma _{\log b(t)}\approx 0.31$.

2 $$\begin{aligned} p(\log b(t)) = \frac{1}{\sqrt{2}\sigma }\exp \left( -\frac{\sqrt{2}|\log b(t)-\mu |}{\sigma }\right) \end{aligned}$$

where $\mu$ and $\sigma$ are the mean and standard deviation of $\log b(t)$, respectively.

To observe detailed properties of the growth rates, we investigate the size dependency by dividing the size of *x*(*t*) into seven intervals growing in powers of 2, i.e.,

$$\begin{aligned} {[}2^0, 2^2), [2^2, 2^4), [2^4, 2^6), [2^6, 2^8), [2^8, 2^{10}), [2^{10}, 2^{12}), [2^{12}, +\infty ). \end{aligned}$$

**Figure 5:**
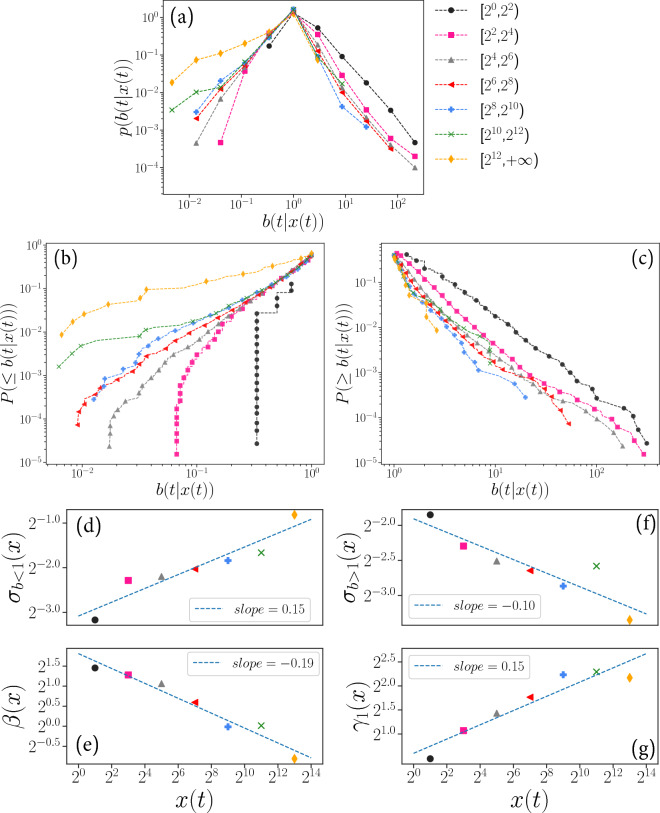
Distribution of *b* (*t* **|** *x* (*t*)) and size-dependent relationship of *b* (*t*) on *x* (*t*). (**a**) Probability density functions of *b*(*t*|*x*(*t*)), where *x*(*t*) is divided into seven non-overlapping intervals, $[2^0, 2^2), [2^2, 2^4), ..., [2^{10}, 2^{12}), [2^{12}, +\infty )$. Different colors and markers are applied to express different intervals of *x*(*t*); (**b**) log-Log plot of cumulative distribution for $b < 1$, where the cumulative probability is calculated by $P(\le b)=\int _0^bp(b')db'$, the maximum probability is $P(b<1)$.; (**c**) log-Log plot of cumulative distribution for $b > 1$, where the cumulative probability is calculated by $P(\ge b) = \int _b^{\infty }p(b')db'$, the maximum probability is $P(b>1)$.; (**d**) estimated size-dependent standard deviations for $b<1$, where $\sigma _{b<1}(x)$ is the standard deviation of $\log b(t)$ with respect to size of *x*(*t*) (**e**) Estimated power law exponent of *b*(*t*|*x*(*t*)) for $b<1$, there the estimates of the power law exponents are stated in the Supplementary Information, and $\beta$ is derived from Eq. (5) in chapter 5.1 of Supplementary Information; (**f**) Estimated size-dependent standard deviations for $b>1$; (**g**) estimated power law exponent of *b*(*t*|*x*(*t*)) for $b>1$, exponent $\gamma _1$ is derived from Eq. (6) in chapter 5.2 of Supplementary Information.

We observe the conditional distribution of *b*(*t*|*x*(*t*)), *p*(*b*(*t*|*x*(*t*))), as shown in Fig. 5a. From these size-dependent growth rate distributions, we find asymmetric behaviors in each interval deviating clearly from the symmetric Laplace distribution.

To clarify more detailed properties of these conditional growth rate distributions, we divide them into growing and shrinking parts, $$b(t|x(t))>1$$ and $$b(t|x(t))<1$$, and plot the cumulative distributions separately to compare the shapes of tails of these distributions. Figure 5b,c show the cumulative distributions for both sides. Figure 5b is the case of $$b(t|x(t))<1$$ and we confirm heavy-tails for $$x(t)>2^2$$ with the larger standard deviation for larger *x* as confirmed in Fig. 5d. The cutoff in the interval $$[2^0, 2^2)$$ is due to the interval range as the smallest growth rate *b*(*t*) in this interval is $$\frac{1}{3}$$. The function form of these distributions is also fitted and is described in chapter 5.1 of Supplementary Information. Figure 5e shows that there is a tendency for the estimated power law exponent to decrease with *x*(*t*). In Fig. 5c the case of $$b(t|x(t))>1$$ is plotted and we find heavy tails in all intervals, and the corresponding standard deviations are smaller for larger *x*, as shown in Fig. 5f. Figure 5g shows that the trend of the power law exponent of the distribution increases with *x*(*t*), where the power law exponents are estimated as described in chapter 5.2 of Supplementary Information. This asymmetric size dependency of the standard deviation is a unique property of the usage of hashtags. In the case of the growth rate of business firms, the standard deviations of growth rate distribution are symmetrically smaller for large firms^25,26^.

### Generalized random multiplicative process model

To take into account the asymmetric size dependence of the growth rate on the usage of hashtags, here, we introduce a new model by generalizing the random multiplicative model. The standard random multiplicative process model is given by

3 $$\begin{aligned} x(t+1) = b(t)x(t) + f(t), \end{aligned}$$

where *b*(*t*) is a growth rate given by an independent and identically distributed (i.i.d.) random variable, and *f*(*t*) is also an i.i.d non-negative random noise.

It is known that^12^ under the condition that $\langle \log b(t) \rangle <0$, the variable *x*(*t*) follows a power law distribution with exponent $\alpha$ which is determined by solving the equation

4 $$\begin{aligned} \langle b(t)^{\alpha }\rangle = 1, \end{aligned}$$

where $\langle \cdot \rangle$ denotes the average.

We generalize the random multiplicative model in the following form:

5 $$\begin{aligned} x(t+1)=b(t|x(t))x(t)+f(t), \end{aligned}$$

where *b*(*t*|*x*(*t*)) is an i.i.d non-negative random variable dependent on the value of *x*(*t*), and *f*(*t*) is an i.i.d non-negative random noise.

Let us consider the case of $x(t)\gg 1$, where we can ignore the random noise *f*(*t*). Then we can approximate Eq. 5 by $x(t+1)\approx b(t|x(t))x(t)$ and the master equation is given as

6 $$\begin{aligned} p(x, t+1) = \int _0^{\infty } d x_1 \int _0^\infty db p(x_1, t)u(b|x_1)\delta (bx_1-x). \end{aligned}$$

Here, $p(\cdot )$ and $u(\cdot )$ are the probability density functions of *x*(*t*) and *b*(*t*|*x*(*t*)), respectively, and $\delta (\cdot )$ is the Dirac delta function.

In Dynamic Properties, we observe that *b* follows different asymmetric distributions with respect to the value of *x* by dividing *x* into seven non-overlapping intervals, $[2^0, 2^2), [2^2, 2^4), [2^4, 2^6), [2^6, 2^8), [2^8, 2^{10}), [2^{10}, 2^{12}), [2^{12}, +\infty )$. Denoting the distribution function of *b* in the *i*th interval, $I_i$, as $u_i(b)$, we assume that in $I_i$, *x* follows a power law distribution with exponent $\alpha _i$, defined as,

7 $$\begin{aligned} p_i(x)=: p(x)\textbf{1}_{x\in I_i} = c_ix^{-\alpha _i - 1}, \ i = 1, 2,..., 6, 7. \end{aligned}$$

Here, $\textbf{1}$ represents the indicator function. Substituting $u_i(b)$ and $p_i(x)$ into Eq. 6 and assuming a stationary solution, we have

8 $$\begin{aligned} p(x) \approx \sum _{i=1}^7\int _{x_1\in I_i} d x_1 \int _0^\infty db c_ix_1^{-\alpha _i-1}u_i(b)\delta (bx_1-x). \end{aligned}$$

Focusing on the case of $x\in I_j$, the probability density function of $p_j(x)$ is given as

9 $$\begin{aligned} p_j(x) \approx \sum _{i=1}^7\int _{x_1\in I_i} d x_1 \int _0^\infty db c_ix_1^{-\alpha _i-1}u_i(b)\delta (bx_1-x)\textbf{1}_{x\in I_j}. \end{aligned}$$

By integrating $x_1$, we have the following equation:

10 $$\begin{aligned} p_j(x) \approx \sum _{i=1}^7 \int _0^\infty db c_i (\frac{x}{b})^{-\alpha _i-1}u_i(b)\frac{1}{b}\textbf{1}_{x\in I_j, x/b \in I_i}. \end{aligned}$$

From the probability density function of *b* for different intervals of *x*(*t*) in Fig. 5a, we observe that *b* takes the value of 1 with high probability, and we make the following approximation in the above equation:

11 $$\begin{aligned} \textbf{1}_{x\in I_j, x/b \in I_i} \approx \textbf{1}_{x\in I_j, x \in I_i} \end{aligned}$$

Thus, Eq. 10 can be approximated as

12 $$\begin{aligned} p_j(x) \approx \int _0^\infty db b^{\alpha _j} u_j(b) c_jx^{-\alpha _j-1} \textbf{1}_{x\in I_j} \end{aligned}$$

Finally, integrating *b*, we have

13 $$\begin{aligned} p_j(x) \approx p_j(x)\langle b_{x\in I_j}^{\alpha _j}\rangle , \end{aligned}$$

which means that we can estimate the power law exponent of *x* in the *j*th interval, $\alpha _j$, by solving the following equation with the corresponding distribution of growth rate *b*:

14 $$\begin{aligned} \langle b_{x\in I_j}^{\alpha _j}\rangle = 1. \end{aligned}$$

This equation for estimating the power law exponent is similar to the Equation of prior study^12^, where the only power law exponent of *x* was determined by the growth rate distribution of the whole *x*. Meanwhile, our results emphasize that for different intervals of *x*, a local scale-dependent power law exponent of *x* is approximately determined by the corresponding growth rate distribution. The reason we obtain similar results to the prior study is highly dependent on the approximation that ignores the effect between the intervals of *x*, i.e., Eq. 11.

### Numerical simulation results

To test the theory of our model of Eq. 5, we perform simulation experiments to confirm that our model can reproduce the cumulative distribution function of *x*(*t*). We check the autocorrelation of *logb*(*t*), as shown in Supplementary Fig. 9; for the lag of 1 day, there is a significant negative correlation, while for the lag of more than 2 days, the correlation is almost 0. Numerical simulation is operated with the assumption of our model that the autocorrelation for *b*(*t*) is always 0, i.e., *b*(*t*) is independently distributed in time. The simulation results show that neglecting the autocorrelation of *b*(*t*) has little effect on the numerical simulations.

As mentioned before, we divide *x*(*t*) into seven non-overlapping intervals to represent the dependence of the growth rate *b*(*t*) on *x*(*t*), that is, $[2^0, 2^2), [2^2, 2^4), [2^4, 2^6), [2^6, 2^8), [2^8, 2^{10}), [2^{10}, 2^{12}), [2^{12}, +\infty )$. While operating simulations, to calculate $x(t+1)$ with Eq. 5, the random numbers of *b*(*t*|*x*(*t*)) are needed. We obtain the random number by randomly sampling the *b*(*t*|*x*(*t*)) calculated from the real data. For large enough *t*, such as $2\times 10^5$, we compare the distribution of the simulated *x*(*t*) with the real one. The details of the simulation experiments are described in Methods.

**Figure 6:**
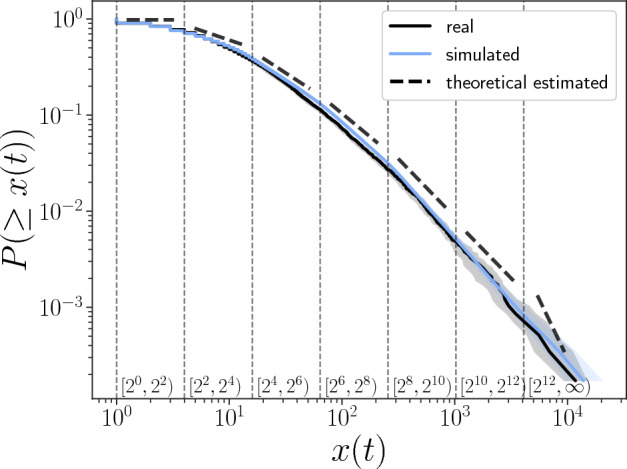
Comparison of cumulative distribution function of simulated *x* (*t*) with the real one. Log-Log plots of CDFs are shown. The black solid line refers to the CDF of *x*(*t*) of real data, and the blue solid line refers to the simulation result of *x*(*t*) where the shadow shows the width between the 25th percentile and the 75th percentile of the simulation results. The vertical dashed lines indicate the boundaries of the interval that divide *x*(*t*); In each interval, the black dashed lines show the theoretical estimation result of the power law exponent.

The results of the simulation are shown in Fig. 6, and we confirm that *x*(*t*) obtained from the simulation reproduces the real distribution well. According to our theory of Eq. 14, the distribution of *b*(*t*) determines the value of the power law exponent, so different intervals of *x*(*t*) correspond to the different power law exponents. We verify that Eq. 14 estimates the value of the scale-dependent power law exponent nicely. We plot the theoretically estimated $\alpha _i$ in slope form in Fig. 6 with the black dashed line. Comparing it to the slope of *x*(*t*) of the real data, we confirm that the theoretical estimation is close to that of the real one. Table 1 gives the theoretical estimation results of $\alpha _i$, finding that $\alpha _i$ changes with the size of *x*(*t*).

**Table 1:** Theoretical estimation of $\alpha$ for different size intervals of *x*(*t*).

Interval of *x*(*t*)	Estimation of $\alpha _i(x)$
$$[2^0, 2^2)$$	0 ± 0
$$[2^2, 2^4)$$	0.310 ± 0.004
$$[2^4, 2^6)$$	0.760 ± 0.005
$$[2^6, 2^8)$$	0.972 ± 0.005
$$[2^8, 2^{10})$$	1.210 ± 0.009
$$[2^{10}, 2^{12})$$	1.229 ± 0.018
$[2^{12}, +\infty )$	2.682 ± 0.058

*x*(*t*) is divided for the case of $division_7$ in Table 2. The estimation is calculated by solving Eq. 14; the positive and negative errors are the estimated errors of the different time series obtained by simulation.

It should be noted that in the interval $$[2^0,2^2)$$, the theoretical estimation of the power law exponent is 0; this is because the distribution generated in this interval is not stationary. It is known that the stationary condition of the random multiplicative process of Eq. 3 is $$\langle \log b(t)\rangle <0$$. This is explained in the following way. We can show that the function $$M(\alpha ) = \langle b(t)^{\alpha } \rangle $$ is convex by calculating its second derivative and $$M(0)=1$$. Thus if $$\langle \log b(t)\rangle $$, which is the first-order differential of $$M(\alpha )$$ at $$\alpha = 0$$, is not smaller than 0, the equation $$\langle b(t)^{\alpha }\rangle = 1$$ does not have a solution in the range $$\alpha >0$$.

**Figure 7:**
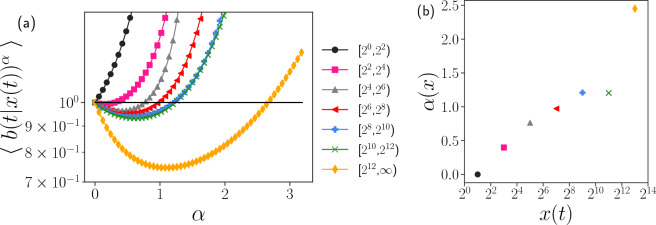
Theoretical estimation of power law exponents. (**a**) For different size intervals of *x*(*t*), corresponding $\langle b(t|x(t))^{\alpha }\rangle$ is plotted as a function of $\alpha$. The intersection of the function and horizontal line with the value of 1 is the theoretical estimation value of the power law exponent. (**b**) Variation of the theoretically estimated value of $\alpha$ with size interval *x*(*t*).

For *x*(*t*) in different size intervals the function curves of $$M(\alpha ) = \langle b^{\alpha }_{x\in I_i}\rangle $$ calculated from our data are shown in Fig. 7a. We observe that they are convex functions and the shape of the functions changes as the size interval of *x*(*t*) changes. The intersection of the functions and horizontal line with value 1 is the theoretical value of the power law exponent. In the interval of $$[2^0,2^2)$$, we observe that the function curve increases from $$\alpha =0$$, which means $$\langle \log b_{x\in [2^0, 2^2)} \rangle >0$$ and there is no power law solution. The variation of the theoretical value with the size of *x*(*t*) is shown in Fig. 7b.

### Results for different divisions

**Figure 8:**
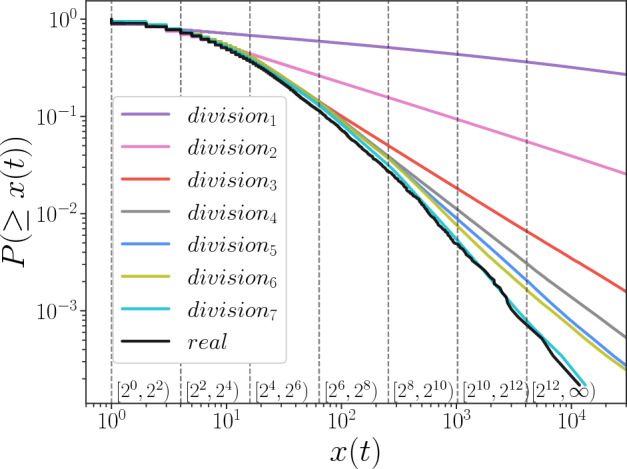
Comparison of the cumulative distribution function for different dividing sizes. The ways of divisions are shown in Table 2. The black line shows the CDF of *x*(*t*) from real data, as in Fig. 2; the other lines refer to the simulation results. The vertical dashed lines indicate the boundaries of the interval that divide the size of *x*(*t*).

To illustrate the influence of the size dependency relationship by numerical simulations, we apply seven different types of interval division of *x*(*t*), as shown in Table 2. For example, $devision_1$ represents the case of *x*(*t*) is not divided, while in other cases, *x*(*t*) is divided into intervals from two to seven. The simulation results are shown in Fig. 8, where the black line is the cumulative distribution from the real data *x*(*t*), and the other colored lines are the distributions obtained from the simulations. We find that all simulated *x*(*t*) follow fat-tailed distributions, but the slopes are different according to the division of *x*(*t*). It is confirmed that the simulation result from $division_1$, which is the case of not dividing *x*(*t*), is the farthest from the real distribution. As the number of dividing intervals increases, the simulation results become closer to the real one, and $division_7$ has the best fitting result. This experimental result verifies the correctness of reproducing the fat-tailed distribution of *x*(*t*) based on our model.

More simulation results for different interval partitioning methods are presented in Supplementary Chapter 3, along with a quantitative evaluation of these results. In Supplementary Chapter 4, the robustness of the simulation results concerning random seeds is described.

**Table 2:** Interval division methods for *x*(*t*) and value of the optimized parameter $\lambda$ of Poisson distribution in our model.

	Methods of dividing intervals of *x*(*t*)	$\lambda$
$Division_1$	$[2^0, +\infty )$	1.3
$Division_2$	$[2^0, 2^2), [2^2, +\infty )$	2.4
$Division_3$	$[2^0, 2^2), [2^2, 2^4), [2^4, +\infty )$	8.2
$Division_4$	$[2^0, 2^2), [2^2, 2^4), [2^4, 2^6), [2^6, +\infty )$	14.6
$Division_5$	$[2^0, 2^2), [2^2, 2^4), [2^4, 2^6), [2^6, 2^8), [2^8, +\infty )$	15.0
$Division_6$	$[2^0, 2^2), [2^2, 2^4), [2^4, 2^6), [2^6, 2^8), [2^8, 2^{10}), [2^{10}, +\infty )$	15.3
$Division_7$	$[2^0, 2^2), [2^2, 2^4), [2^4, 2^6), [2^6, 2^8), [2^8, 2^{10}), [2^{10}, 2^{12}), [2^{12}, +\infty )$	14.9

The optimization method for $\lambda$ is described in Methods.

## Methods

### Details of simulation

To test the theory of our generalized random multiplicative process model in Eq. (5), we perform simulation experiments to observe the distribution of the generated *x*(*t*). Here, we explain more details about the simulation. Firstly, three variables in the model are defined as follows:

- $\varvec{x}(\varvec{t})$: *x*(*t*) is set to take values of positive integers, as in the real data of hashtag usage count, which takes positive integers.
- $\varvec{b}(\varvec{t}|\varvec{x}(\varvec{t}))$: *b*(*t*|*x*(*t*)) is an i.i.d. random variable; the distribution of *b*(*t*|*x*(*t*)) changes depending on the choice of the interval of *x*(*t*). While operating simulation, *b*(*t*|*x*(*t*)) is randomly sampled from the real data.
- $\varvec{f}(\varvec{t})$: Random noise *f*(*t*) is set to follow a Poisson distribution with mean $\lambda$, i.e., $f(t)\overset{i.i.d.}{\sim }Po(\lambda )$. *f*(*t*) is only added when $b(t|x(t))x(t) < 1$; this means that whenever *x* decreases close to 0 at time *t*, we produce a random value that follows the Poisson distribution at time $t+1$, which corresponds to a rebirth of a new hashtag.
  We optimize the parameter $\lambda$ by minimizing the Kolmogorov-Smirnov distance between the distribution of *x*(*t*) obtained from the simulation and real data, i.e.,
  15 $$\begin{aligned} Minimize \ \ D_{KS}(\lambda ) = sup_x|F_{simualation}(x, \lambda )-F_{real}(x)| \end{aligned}$$
  where $F_{simualation}(\cdot )$ and $F_{real}(\cdot )$ refer to the cumulative distribution functions of simulation and real data, respectively. The optimization is done by grid search. The results for each division are shown in Table 2.

We simulate 5805 time series of $x_i(t), i=1,\dots ,5805$ independently, as many as the hashtag series of the real data. The initial values, $x_i(0)$, are random numbers that follow a uniform distribution range from 1 to 100, 000. Setting the maximum time *t* to 200, 000, we perform the simulation. The algorithm for the simulation is shown below. The distribution of $x_i(200,000), i=1,\dots ,5805$ is compared with that from the real data.

**Algorithm 1:**
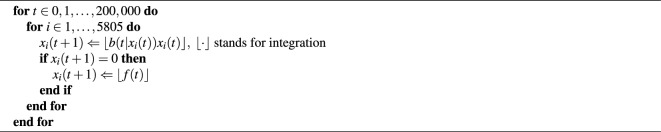
Simulation of the model of $x(t+1)=b(t|x(t))x(t)+f(t)$.

## Conclusion and discussion

In this paper, we analyzed a fat-tailed distribution in the time series data of hashtag usage count on Weibo by analyzing the growth rate of these series, and we observed that there is a clear size dependence between the growth rate of hashtag usage and usage count. As a model of hashtag usage count, we introduced a new model of a size-dependent random multiplicative process and theoretically and numerically proved that a fat-tailed distribution with a size-dependent power law exponent is generated. We derived Eq. (14) which enables us to estimate the size-dependent power law exponents from the growth rates in the same interval. By conducting numerical simulations, we confirmed that our model reproduces the whole shape of the fat-tailed distribution of hashtag usage count nicely.

From a physical perspective, the dynamics of the usage count for a single hashtag *x*(*t*) follows a discrete Langevin equation described by Eq. 3, capturing the randomness of growth rate of hashtag usage count. The multiplicative noise term, *b*(*t*), represents the growth rate. It is caused by factors such as user posting activities, interactions between hashtags, and other potential influences. Taking into account the size dependence of *b*(*t*) and *x*(*t*) observed from the data, we refined the model to Eq. 5. From the statistical properties of *b*(*t*|*x*(*t*)) in Fig. 5, it can be discerned that as *x*(*t*) increases, the diffusion or spread of *x*(*t*) becomes more restricted. This highlights the distinct behavior of popular hashtags, and we’ve incorporated these characteristics into our model.

Few studies have investigated the modeling of the appearance frequency of hashtags from the perspective of complex systems, so we believe that there is great potential for the development of an analysis of the appearance frequency of hashtags based on our model. We give three possible developments of our study. Firstly, in this paper, we focused on the hashtags which were used every day during the observation period and therefore, we need other models to illustrate the properties of hashtags that are not used every day. Secondly, we assume that there is no auto-correlation in growth rates in our proposed model, so the model should be extended if the appearance frequency of hashtags has auto-correlation. Thirdly, our model is primarily an approximation of the mesoscopic dynamics encompassing hashtag features, we are in the process of leveraging this foundational model to bridge the gap with a micro-level perspective, potentially leading to the development of an agent-based model.

Fat-tailed distributions are widely observed in natural and social phenomena. When we observed it, we tend to characterize the distribution with a power law distribution with just one power law exponent as shown in Fig. 2. However, by the analyses of the generalized random multiplicative process, it is numerically and theoretically clarified that the distribution is fat-tailed with the size-dependent power law exponent if the growth rate of the variable has size dependency. Note that not only our model can explain the observed fat-tailed distribution, other theories such as maximization of Tsallis entropy^4^ or superstatistical model^5–7^ which works well with turbulent time series can also be applicable. We show that a q-exponential distribution fits well with our data in chapter 2 of Supplementary Information.

Recently we applied our model to the sales of firms and bacterial count of each species in the intestine ecosystems and observed that size changes over time without auto-correlation and follows a fat-tailed distribution from both data sets. We believe our research can be applied to various phenomena of nature and social systems.

### Supplementary Information

Supplementary Information.

## Data Availability

The datasets used in the current study are not accessible to the public because of Weibo’s open API policy, which prioritizes the confidentiality of personal data. However, aggregated and anonymized versions of the data can be obtained by contacting the corresponding author and making a reasonable request. If you are interested in acquiring similar data, you can utilize the Weibo API (https://open.weibo.com/wiki/API). More information and specifics can be found in chapter 1 of Supplementary Information.
