# Supplementary material for: Scale-dependent power law properties in hashtag usage time series of Weibo
Source: Sci Rep. 2023 Dec 15;13:22298. doi: 10.1038/s41598-023-49572-6 (PMC10724262; doi:10.1038/s41598-023-49572-6)
Supplement: Supplementary file 1 — Supplementary Information. [file 41598_2023_49572_MOESM1_ESM.pdf]

Scale-dependent Power Law Properties in Hashtag Usage Time Series of Weibo

Jiwei J. Jiang, Kenta Yamada, Hideki Takayasu, Misako Takayasu

1 Data Set

Weibo is a mainstream micro-blog social media in China, we collect Weibo data through the publicly available API. Due to the huge volume of users and the limitation of the API, it is impossible to collect all the data for analysis, so we focus on the hashtag posting behavior of sampled users with a total number of about 300,000. We collected a total of about 20 million micro-blogs from these users posted between July 21st and August 18th in 2021, and extracted about 600,000 different hashtags. We chose the 5805 hashtags that are posted every day during the observation interval (29 days) as the object for the convenience of the analysis. The detailed process of collecting data is shown in the flow chart in Figure 1.

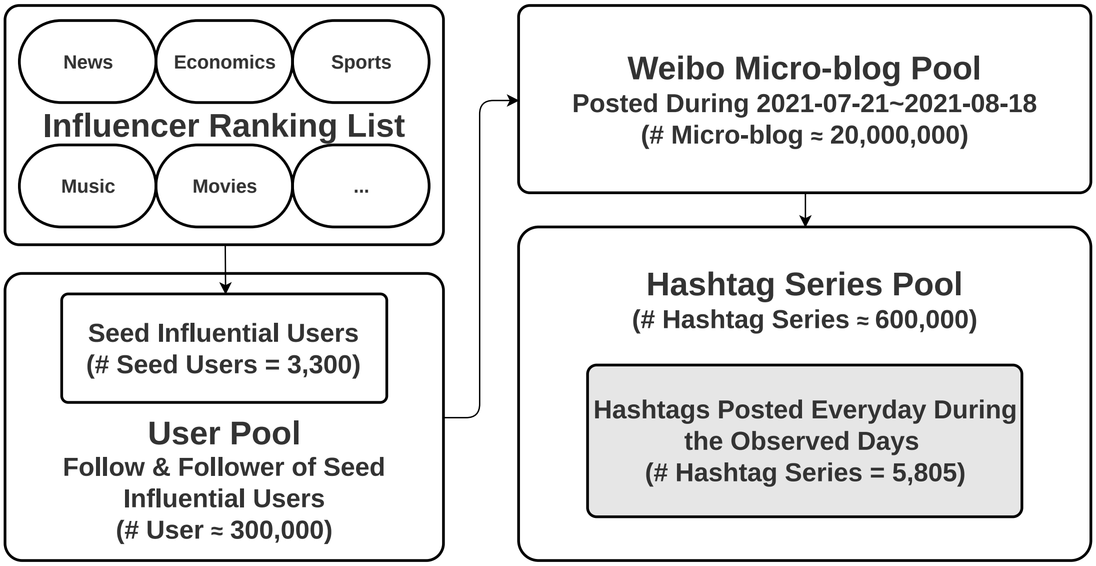

Supplementary Figure 1: **Flowchart for collecting the hashtag usage count time series with open API**. Due to the huge volume of users and the limitation of the API, it is impossible to collect all the data, and we focus on the hashtags posted by sampled users. We obtain the data in three steps, first, we select 3,300 seed users from the publicly influential users' ranking lists which contain the official top 100 influences in 33 different fields. The following and followers of the seed users are sampled, getting a sample of about 300,000 users which we called a user pool; Then, from these users, we collected micro-blogs posted between July 21st and August 18th in 2021 to get our Weibo micro-blog Pool, a total of about 20 million blogs; Finally, we extract hashtag data from micro-blogs to get the hashtag pool, where about 600,000 different hashtags are extracted. The duration of hashtags ranges from 1 day to 29 days (the longest observation interval). For the convenience of the analysis, we choose 5805 hashtags that were used every day during the observation interval (29 days) as the object of analysis.

2 Fitting of Hashtag Usage Distribution with q-exponential

We fit hashtag usage distribution shown in fig 3 of main paper by q-exponential distribution. The function form of pdf is used as follows,

$$p_q(x) = p_0[1-(1-q)\frac{x}{x_0}]^{1/(1-q)} \tag{1}$$

where $1-(1-q)x/x_0 > 0$ and $p_0 = (2-q)/x_0$. The function of cdf is as follows,

$$P(\geq p_q(x)) = [1-(1-q')\frac{x}{x'_0}]^{1/(1-q')} \tag{2}$$

where $q < 2$ and $q' = 1/(2-q), x'_0 = x_0/(2-q)$ and $p'_0 = p_0x_0/(2-q)$.

While fitting, the parameter q and x_0 are determined by grid search, and result shows that $q = 1.45, x_0 = 5.9$. The fitting result is shown in Supplementary Figure 2 below, the black dashed line refers to the line of power law exponent equals to 1.22 which is calculated by $1/(1-q')$.

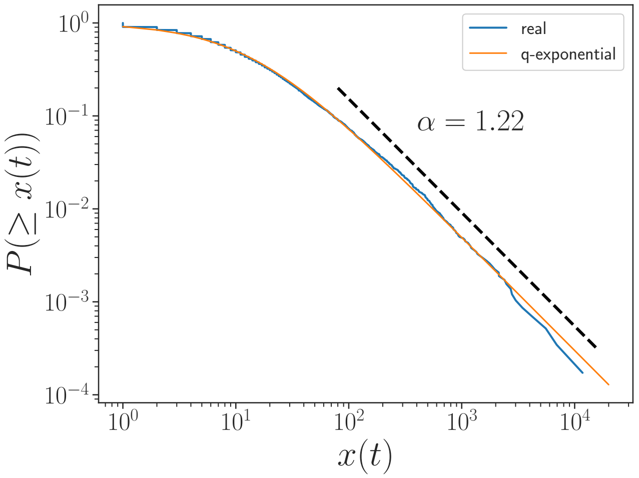

Supplementary Figure 2: **Fitting of hashtag usage distribution with q-exponential**. As legend shows, distribution of "real" is from data, and is the same distribution as figure 3 of main paper. Distribution of "q-exponential" is from Equation (2), where $q = 1.45, x_0 = 5.9$.

3 Evaluation of the Simulated Results

Differing from the interval partitioning method in Table 2 of the main paper, here we provide simulations for intervals evenly partitioned on a logarithmic scale, for any given number of intervals. Specifically, with a given number of intervals N and the maximum observed value of x in the actual data, x_{max}, we divide the intervals as $[base^0, base^1), [base^1, base^2), ..., [base^{N-1}, X_{max})$. We calculate the value of base with $base^N = x_{max}$ to obtain the partition for any given number of intervals. Finally, because in the simulation, x can take on values without an upper limit, we set the Nth interval as $[base^{N-1}, +\infty)$ instead of $[base^{N-1}, x_{max})$. The values of base and the interval partitioning method are shown in Supplementary Table 1 below. The simulation results are shown in Supplementary Figure 3.

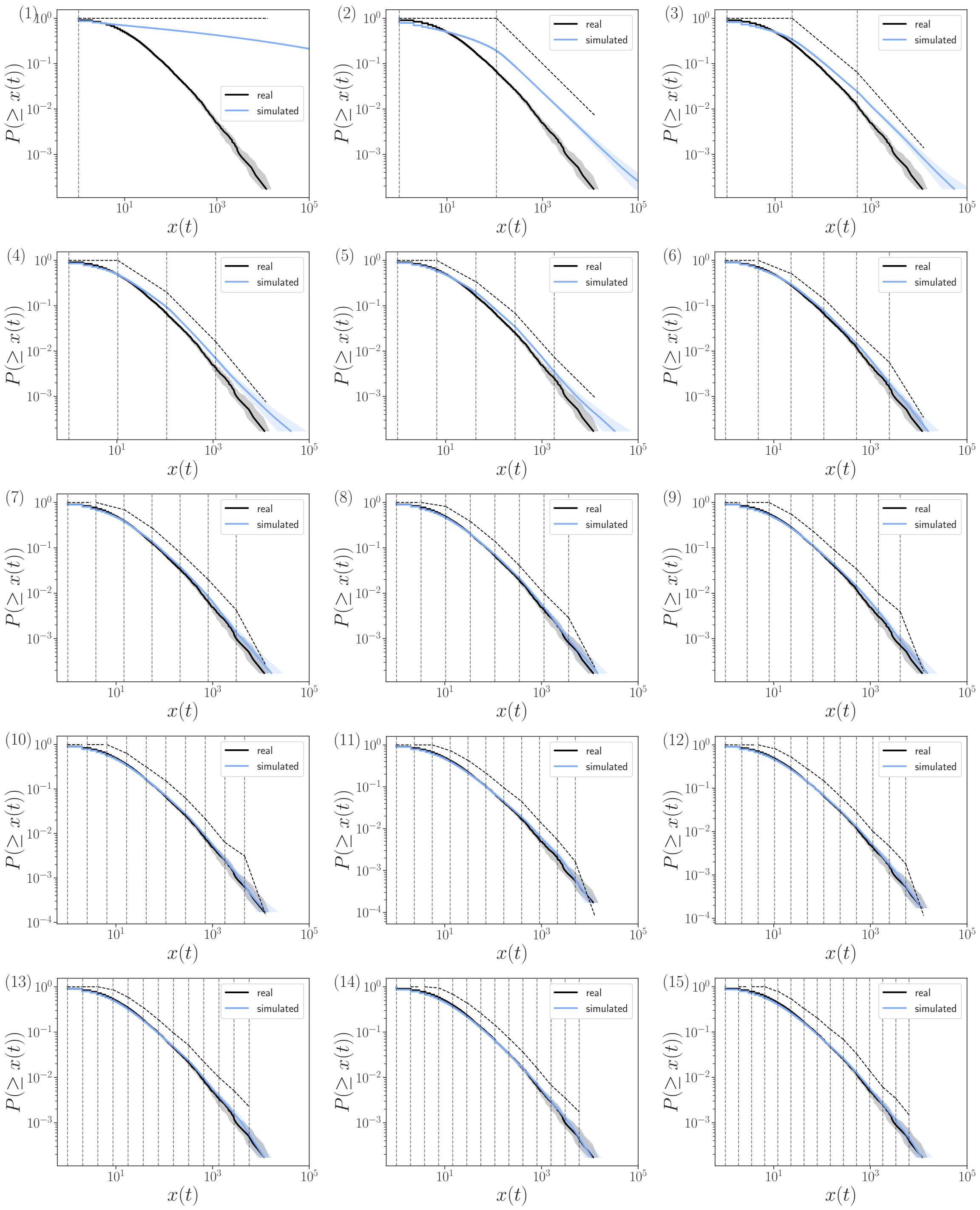

Supplementary Figure 3: **Comparison of cumulative distribution function of simulated $x(t)$ with the real one.** Log-Log plots of CDFs are shown. The black solid line refers to the CDF of $x(t)$ of real data, and the blue solid line refers to the simulation result of $x(t)$ where the shadow shows the width between the 25th percentile and the 75th percentile of the simulation results. The vertical dashed lines indicate the boundaries of the interval that divide $x(t)$; In each interval, the black dashed lines show the theoretical estimation result of the power law exponent.

Supplementary Table 1: **Interval division methods for $x(t)$.**

N	$base = e^{X_{max}/N}$	Partitioning method of $x(t)$
1	1	$[base^0, +\infty)$
2	108.416	$[base^0, base^1), [base^1, +\infty)$
3	22.737	$[base^0, base^1), [base^1, base^2), [base^2, +\infty)$
4	10.412	$[base^0, base^1), [base^1, base^2), ..., [base^3, +\infty)$
5	6.517	$[base^0, base^1), [base^1, base^2), ..., [base^4, +\infty)$
6	4.768	$[base^0, base^1), [base^1, base^2), ..., [base^5, +\infty)$
7	3.815	$[base^0, base^1), [base^1, base^2), ..., [base^6, +\infty)$
8	3.227	$[base^0, base^1), [base^1, base^2), ..., [base^7, +\infty)$
9	2.833	$[base^0, base^1), [base^1, base^2), ..., [base^8, +\infty)$
10	2.553	$[base^0, base^1), [base^1, base^2), ..., [base^9, +\infty)$
11	2.344	$[base^0, base^1), [base^1, base^2), ..., [base^{10}, +\infty)$
12	2.184	$[base^0, base^1), [base^1, base^2), ..., [base^{11}, +\infty)$
13	2.056	$[base^0, base^1), [base^1, base^2), ..., [base^{12}, +\infty)$
14	1.953	$[base^0, base^1), [base^1, base^2), ..., [base^{13}, +\infty)$
15	1.868	$[base^0, base^1), [base^1, base^2), ..., [base^{14}, +\infty)$

We measure the error between real data and simulated data using the Mean Absolute Error (MAE) of the logarithmic values of PDF, which is defined as follows,

$$MAE = \sum_{i=1}^{M} \frac{|\log p(x_i) - \log p(\hat{x}_i)|}{M} \tag{3}$$

where $p(x_i)$ and $p(\hat{x}_i)$ refers to PDF value of bin i of real data and simulated data, M refers to the bin size. As shown in Supplementary Figure 4, we observe that as interval count increases, the MAE values gradually decrease and stabilize after reaching a number of 8 intervals. At this point, we consider the number of interval partitions to be enough good, and the estimation of power-law exponents is also reasonably accurate. Note that a larger interval count is not necessarily better, as with an increasing number of interval partitions, the accuracy of power-law exponent estimation decreases due to a decrease in the data volume within each interval, especially for intervals with larger values of x.

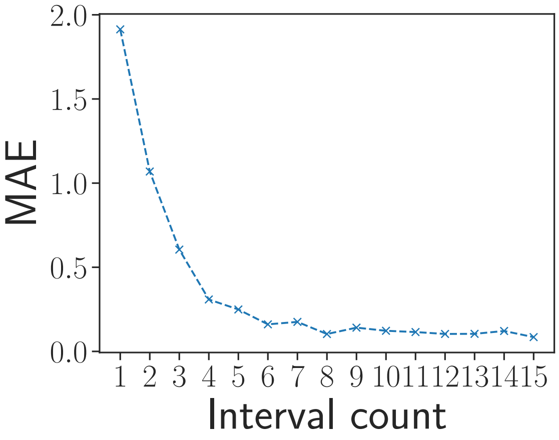

Supplementary Figure 4: **Mean Absolute Error (MAE) of simulated result.** MAE is defined by Equation 3.

4 The robustness of the simulation results

Supplementary Figure 5 represents the simulation results using different random number seed to select $b(t|x(t))$ from growth rate in real data. In this case, we divide $x(t)$ into 7 areas, $[2^0, 2^2), ..., [2^{12}, +\infty)$, labeled as "devision7" in our paper. The distributions are stationary and almost the same within areas that have enough samples.

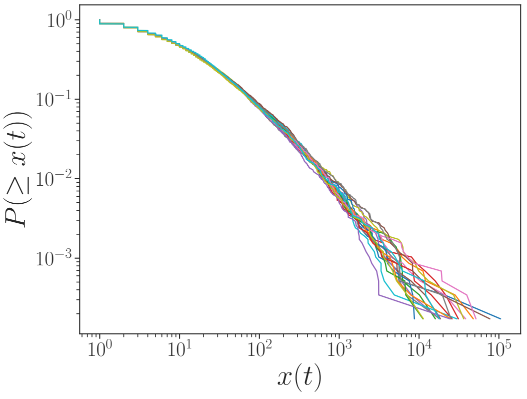

Supplementary Figure 5: Simulaiton results using different random number seed to select $b(t|x(t))$ from real data. Log-Log plots of CDFs are shown with different color.

5 Functional Form of the Growth Rate Distributions

In the chapter on Dynamic Properties of main text, we observed that the logarithmic growth rate $\log b(t) = \log \dfrac{x(t+1)}{x(t)}$ follows the Laplace distribution as shown in Figure 4, the Laplace distribution also known as double exponential distribution, which means that $\log b$ follows exponential distribution on both sides of greater than μ and less than μ, respectively. Thus the cumulative probability density function for b is approximately power law function as follows.

$$\begin{cases} P(\leq b) \propto b^{\sqrt{2}/\ \sigma}, b \leq e^{\mu} \approx 1 \\ P(> b) \propto b^{-\sqrt{2}/\ \sigma}, b > e^{\mu} \approx 1 \end{cases} \tag{4}$$

Here $\mu \approx 0$, $\sigma \approx 0.31$ refer to the mean and the standard deviation value of $\log b(t)$. In Figure 5 (a) we demonstrated that the distributions of $b(t|x(t))$ for different intervals of $x(t)$ are asymmetric and deviate clearly from the Laplace distribution. For the cumulative distributions of $b(t|x(t)) < 1$ and $b(t|x(t)) > 1$ which are shown in Figure 5 (b)(c), the distributions at log-log scale are approximately straight lines, implies that these distributions can still be approximated by power law functions however the power law exponents are deviated from that in Equation 4. Here we fit these cumulative distributions.

5.1 Cumulative distribution functions of $b(t|x(t)) < 1$

For this case, we observe in Figure 5 (b) that for different sizes of $x(t)$, $b(t)$ is a power law like distribution with cutoff. Since it is not often to fit a power law distribution with a cumulative probability calculated by $P(\leq b) = \int_0^b p(b')db'$, we first observe and fit the cumulative probability function of $m = \dfrac{1}{b(t|x(t))}$, which exhibits a cumulative probability function $P(\geq m)$ symmetric to $P(\leq b)$, as shown in Supplementary Figure 6.

Since for different intervals of $x(t)$, $m = \dfrac{x(t)}{x(t+1)}$, and the maximum value of m takes the maximum value of $x(t)$ in that interval when $x(t+1) = 1$, so we consider fitting the distribution of m as a power law function with specific cutoff, as follows.

$$P(\geq m) \propto (m + m_0)^{-\beta} - (m_c + m_0)^{-\beta},\ 1 < m \leq m_c \tag{5}$$

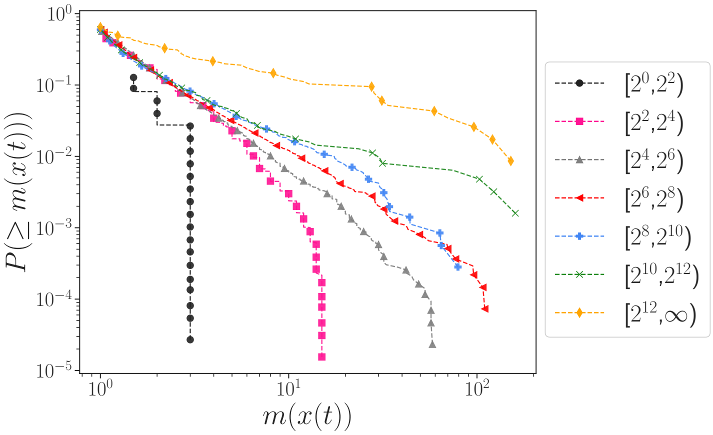

Supplementary Figure 6: **Cumulative distribution of** $m(x(t))$**.** Log-Log plot of cumulative distribution for m, m is calculated by inverse of growth rate $\frac{1}{b(t|x(t))}$ where $b(t|x(t)) < 1$. The cumulative probability is calculated by $P(\geq m) = \int_m^\infty p(m')dm'$.

where m_c is the parameter that controls the position of cutoff, such that $P(\geq m_c) = 0$. For different interval of $x(t)$, m_c is set to maximum value of $x(t)$ minus 1 as shown in Supplementary Table 2. The other two parameters m_0 and β are estimated by the maximum likelihood method. The estimated parameters for each interval of $x(t)$ are presented in Supplementary Table 2. After approximating the functional form of the cumulative distribution of m, we can obtain the functional form of the cumulative distribution of $b(t|x(t))$ as follows.

$$P(\leq b) = P(\geq \frac{1}{b}) = P(\geq m) \propto (\frac{1}{b} + m_0)^{-\beta} - (m_c + m_0)^{-\beta},\ \frac{1}{m_c} \leq b < 1 \tag{6}$$

The three parameters are the same as the cumulative distribution of m, as shown in Supplementary Table 2. Supplementary Figure 7 shows the fitting result of $b(t|x(t))$.

Supplementary Table 2: **Estimated result of the parameters of** $b(t|x(t))$ **for the case of** $b(t|x(t)) < 1$**.**

⟍	$[2^0,2^2)$	$[2^2,2^4)$	$[2^4,2^6)$	$[2^6,2^8)$	$[2^8,2^{10})$	$[2^{10},2^{12})$	$[2^{12},+\infty)$
m_0	0.60	0.53	0.11	-0.36	-0.72	-0.68	-0.56
β	2.74	2.43	2.09	1.51	0.99	1.01	0.57
m_c	2^2-1	2^4-1	2^6-1	2^8-1	$2^{10}-1$	$2^{12}-1$	∞

5.2 Cumulative Distribution Functions of $b(t|x(t)) > 1$

For this case, we find that for different intervals of $x(t)$, $b(t)$ is a power law like distribution, and we find that the power law exponent changes for large value $b(t)$ as shown in Supplementary Figure 8, we consider the following functional form to fit the distribution,

$$P(\geq b) \propto \begin{cases} b^{-\gamma_1} & ,1 < b < b_{mid} \\ b^{-\gamma_2} & ,b \geq b_{mid} \end{cases} \tag{7}$$

where b_{mid}, γ_1 and γ_2 are parameters, we obtain the estimation of the three parameters by minimizing the sum of squared errors in the log-log scale. The results are presented in Supplementary Table 3. The distributions of these function form is shown in Supplementary Figure 8.

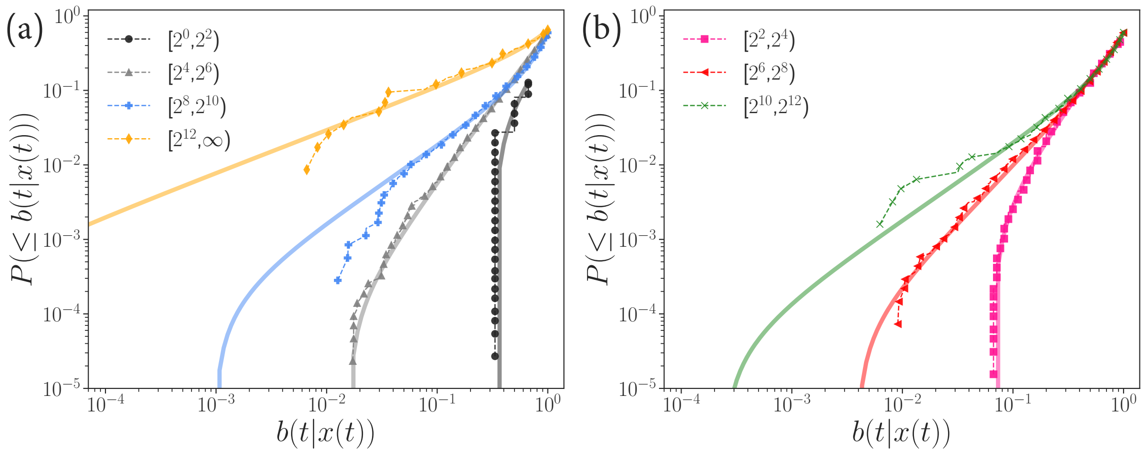

Supplementary Figure 7: **The fitting results of the cumulative distribution of** $b(t|x(t))$ **for the case of** $b(t|x(t)) < 1$**.** The lines with graphs are of real data, the solid lines are of fitted function forms from Equation 6. **(a)** Distributions for the case of $x(t)$ in intervals of $[2^0, 2^2)$, $[2^4, 2^6)$, $[2^8, 2^{10})$ and $[2^{12}, +\infty)$; **(b)** Distributions for the case of $x(t)$ in intervals of $[2^2, 2^4)$, $[2^6, 2^8)$ and $[2^{10}, 2^{12})$.

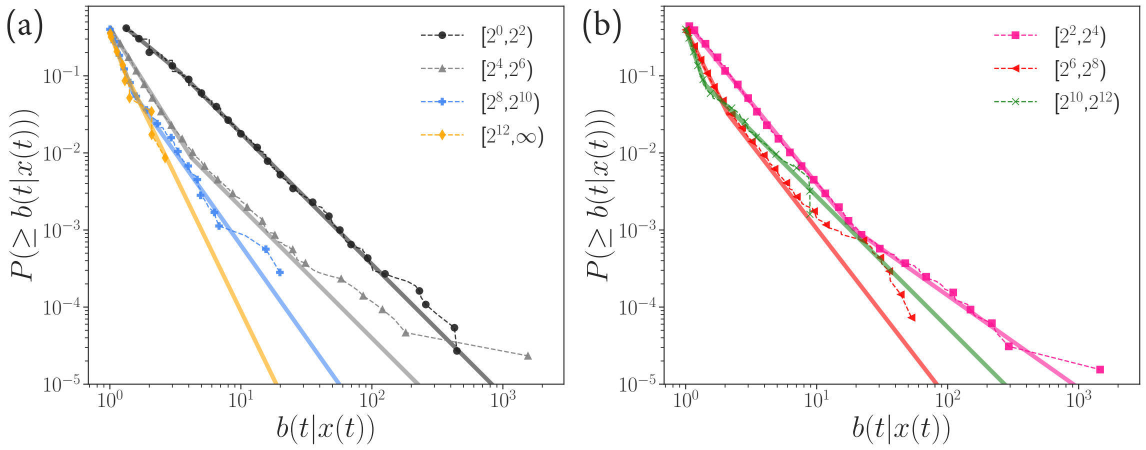

Supplementary Figure 8: **The fitting results of the cumulative distribution of** $b(t|x(t))$ **for the case of** $b(t|x(t)) < 1$**.** The lines with graphs are of real data, the solid lines are of fitted function forms from Equation 7. **(a)** Distributions for the case of $x(t)$ in intervals of $[2^0, 2^2)$, $[2^4, 2^6)$, $[2^8, 2^{10})$ and $[2^{12}, +\infty)$; **(b)** Distributions for the case of $x(t)$ in intervals of $[2^2, 2^4)$, $[2^6, 2^8)$ and $[2^{10}, 2^{12})$.

Supplementary Table 3: **Estimated result of the parameters of** $b(t|x(t))$ **for the case of** $b(t|x(t)) > 1$**.**

	$[2^0, 2^2)$	$[2^2, 2^4)$	$[2^4, 2^6)$	$[2^6, 2^8)$	$[2^8, 2^{10})$	$[2^{10}, 2^{12})$	$[2^{12}, +\infty)$
b_{mid}	3.5	19.1	4.2	2.1	1.5	1.4	1.3
γ_1	1.4	2.1	2.7	3.4	4.7	4.9	4.5
γ_2	1.7	1.2	1.7	2.2	2.4	1.7	3.5

6 Auto-correlation function (ACF) of Growth Rate

In the Numerical Simulation Results section of the main text, we describe that the logarithmic growth rate, $\log b(t)$, exhibits a negative auto-correlation at a time lag of 1 day, and for the time lag greater than one day, the auto-correlation coefficient is almost 0, as shown in Supplementary Figure 9 below.

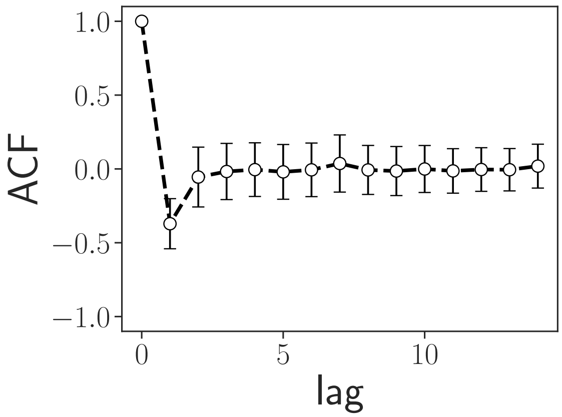

Supplementary Figure 9: **Auto-correlation function of** $\log b(t)$. For all time series $x_i(t)$, the mean value of auto-correlation functions of $\log b_i(t) = \log \frac{x_i(t+1)}{x_i(t)}$ is plotted, and the error bar indicates the standard deviation.

Although we neglected the auto-correlation in the numerical simulations, we add here more details and examples. As shown in Supplementary Figure 10 below, we plot the scatter plot of the size of $\log b$ at time t and time $t+1$. Compare to the plot between time t and time $t+2$, we can find that there is a negative correlation of $\log b(t)$ at the time lag of 1. The scatter plots with a lag greater than 2 are similar to that with a lag of 2 and are therefore omitted here.

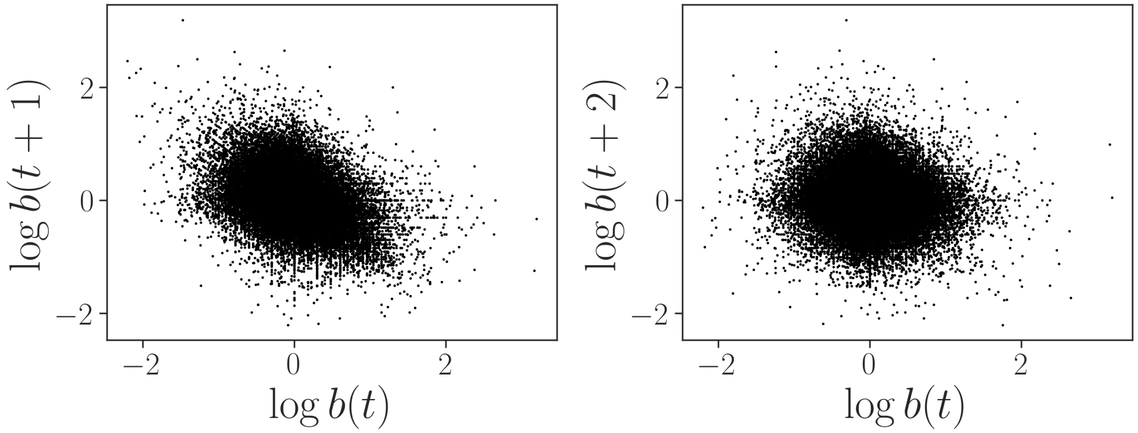

Supplementary Figure 10: **Scatter plot of** $\log b(t)$ **with** $\log b(t + \textbf{lag})$. The graphs with lags of 1 and 2 are plotted. The distribution with a lag greater than 2 is similar to the distribution with lag a of 2 and is therefore omitted.

This negative auto-correlation at the lag of 1 day indicates a tendency for the variable $\log b(t)$ to decrease immediately after an increase, and vice versa. We selected hashtags for which the auto-correlation coefficient of lag 1 day is less than 0.5, and observe the time series of the hashtag usage count, $x(t)$, and growth rate, $log(b(t))$. The time series of the number of users of the corresponding hashtags and their growth rate of them are also observed. Typical three examples are given in Supplementary Figure 11.

- (a) (b) (c) shows the time series of the hashtag '#Photography'

As shown in (c), the ACF of the growth rate of the number of users who use this hashtag is similar to the ACF of the growth rate of the hashtag usage count, and we infer that the negative auto-correlation of lag one day of $\log b(t)$ is due to the negative auto-correlation of the growth rate of the number of users.

- (d) (e) (f) show the time series of the hashtag '#Jujutsu Kaisen'

  Hashtag '#Jujutsu Kaisen' is the name of a Japanese anime, from the time series of $x(t)$ in (d), it exhibits a clear feature of spikes, which we inferred is the reason for the negative auto-correlation of lag 1 day of $\log b(t)$.

- (g) (h) (i) show the time series of hashtag '#Career Recruitment'

  As shown in the time series of the number of users of this hashtag in Figure (g), this is a hashtag used by only one user, and the number of daily usages is often stable, with a sudden increase in usage count on individual days, often accompanied by a decrease in submissions on the next day, which we inferred is the reason for the negative auto-correlation of lag 1 day of $\log b(t)$.

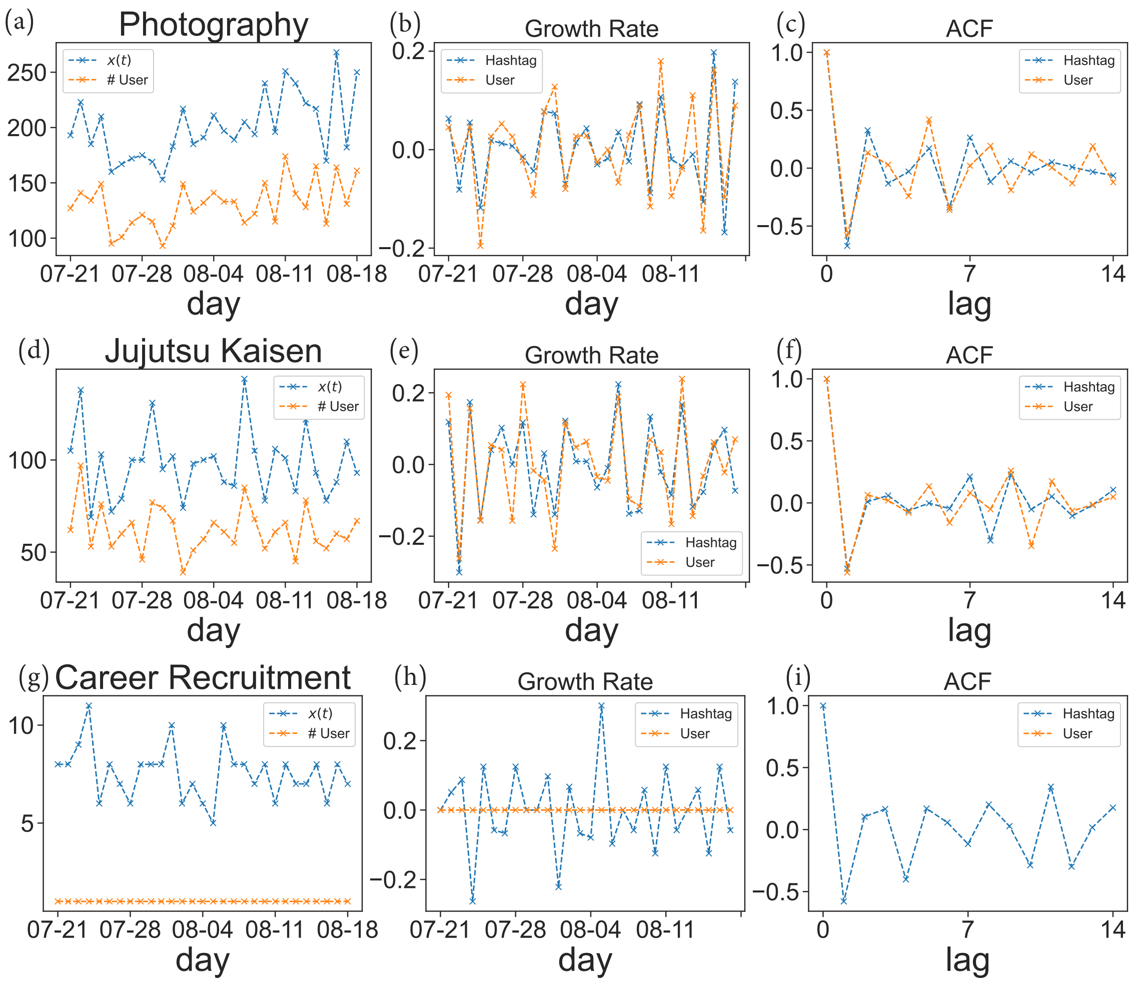

Supplementary Figure 11: **Time series and ACF examples**. Three examples of different hashtags are listed in different rows. The blue dotted lines represent hashtags, the first column is the daily usage count time series of hashtags, the second column is the time series of the corresponding growth rate, and the third column is the ACF of the growth rate. The orange dotted lines represent the number of users, the first column is the time series of the number of users of the corresponding hashtag, the second column is the corresponding growth rate, and the third column is the ACF.**(a)(b)(c)** Hashtag of '#Photography'; **(d)(e)(f)** Hashtag of '#Jujutsu Kaisen'; **(g)(h)(i)** Hashtag of '#Career Recruitment'.
